# The deletion of bacterial dynamin and flotillin genes results in pleiotrophic effects on cell division, cell growth and in cell shape maintenance

**DOI:** 10.1186/1471-2180-12-298

**Published:** 2012-12-19

**Authors:** Felix Dempwolff, Hanna M Wischhusen, Mara Specht, Peter L Graumann

**Affiliations:** 1 Mikrobiologie, Fachbereich für Biologie, University of Freiburg, Schänzlestraße 1, Freiburg, 79104, Germany; 2 LOEWE Center for Synthetic Microbiology, SYNMIKRO, Philipps-University Marburg, Marburg, 35043, Germany; 3 BIOSS Centre for Biological Signalling Studies, University of Freiburg, Schänzlestr. 18, Freiburg, 79108, Germany

## Abstract

**Background:**

In eukaryotic cells, dynamin and flotillin are involved in processes such as endocytosis and lipid raft formation, respectively. Dynamin is a GTPase that exerts motor-like activity during the pinching off of vesicles, while flotillins are coiled coil rich membrane proteins with no known enzymatic activity. Bacteria also possess orthologs of both classes of proteins, but their function has been unclear.

**Results:**

We show that deletion of the single *dynA* or *floT* genes lead to no phenotype or a mild defect in septum formation in the case of the *dynA* gene, while *dynA floT* double mutant cells were highly elongated and irregularly shaped, although the MreB cytoskeleton appeared to be normal. DynA colocalizes with FtsZ, and the *dynA* deletion strain shows aberrant FtsZ rings in a subpopulation of cells. The mild division defect of the *dynA* deletion is exacerbated by an additional deletion in *ezrA*, which affects FtsZ ring formation, and also by the deletion of a late division gene (*divIB*), indicating that DynA affects several steps in cell division. *DynA* and *mreB* deletions generated a synthetic defect in cell shape maintenance, showing that MreB and DynA play non-epistatic functions in cell shape maintenance. TIRF microscopy revealed that FloT forms many dynamic membrane assemblies that frequently colocalize with the division septum. The deletion of *dynA* did not change the pattern of localization of FloT, and vice versa, showing that the two proteins play non redundant roles in a variety of cellular processes. Expression of dynamin or flotillin T in eukaryotic S2 cells revealed that both proteins assemble at the cell membrane. While FloT formed patch structures, DynA built up tubulated structures extending away from the cells.

**Conclusions:**

*Bacillus subtilis* dynamin ortholog DynA plays a role during cell division and in cell shape maintenance. It shows a genetic link with flotillin T, with both proteins playing non-redundant functions at the cell membrane, where they assemble even in the absence of any bacterial cofactor.

## Background

The dynamin protein superfamily is a large group of mechanochemical GTPases. Members of this family play an important role in vesicle formation, clathrin-dependent endocytosis, renewal of membrane components, and the division of organelles [1,2]. Dynamin-like proteins have a characteristic arrangement of an N-terminal GTPase domain, a central domain and a GTPase effector domain [3]. Canonical dynamin has two additional domains, a pleckstrin homology domain and a C-terminal proline and arginine-rich domain that mediates interaction with proteins and lipids [4]. The GTPase domain couples GTP hydrolysis with a mechanical reaction that can confer motor-like functions. The middle domain is only poorly conserved and functions in multimerization of dynamin-like proteins. The effector domain serves in stimulation of GTPase activity and in the interaction of dynamin molecules. It contains characteristic heptad repeat regions that can form coiled coils, and which are relevant for dynamin interactions [3,5]. In spite of their similar general arrangement, dynamin-like proteins are highly divergent in their individual setup, probably reflecting the broad spectrum of cellular functions they are involved in [4,6].

The GTPase motifs within the GTPase domain show similarity to regulatory Ras-like GTPases [7], however, the domain is much larger than that of regulatory GTPases, and does not require additional stimulatory proteins, but instead is 100 fold enhanced through oligomerization. The domain displays low GTP affinity (10 to 100 μM), but high GTPase activity. Purified dynamin has been shown to self-organize into rings and helical structures that are able to attach to lipid membranes and to distort them into large tubular structures. Addition of GTP gives rise to a conformational change and to a constriction, which ultimately leads to a fragmentation of the membrane. Some dynamin-like proteins have a high affinity to negatively charged phospholipids [3,4,6], indicating that membrane composition and lipid rafts may be important for the localization of dynamins.

One of the best understood tasks performed by dynamin is pinching off of clathrin-coated vesicles. Dynamin assembles like a collar around clathrin-coated membrane invaginations and through GTP hydrolysis driven conformational change dissects the vesicle from the membrane [8,9]. In addition to this mechanical role, dynamin is discussed to be responsible for recruiting additional factors to the clathrin pits to facilitate and regulate the formation of the vesicles [10].

Interestingly, many bacterial genomes also contain potential dynamin-like proteins. The crystal structure of the protein termed BDLP (bacterial dynamin-like protein) from the filamentous cyanobacterium *Nostoc punctiforme* revealed that indeed, this protein has a typical dynamin GTPase domain, a neck domain, and an end domain [11]. Structural analysis of BDLP suggests that it operates as a homodimer as smallest unit. The purified protein shares several properties with dynamins: it self-assembles into tubular structures containing radial spokes, which tubulate membranes [12]. *In vivo*, BLDP localizes as irregular focus-like assemblies at the cell membrane [11]. *Bacillus subtilis* is a model organism for Gram positive bacteria and contains a predicted dynamin-like protein, DynA. DynA has recently been shown to be able to bind to membranes and to mediate membrane fusion *in vitro*, even in the absence of GTP [13]. However, there is no information yet on the function of bacterial dynamin-like proteins *in vivo*. A possible function in cell division has been proposed [13]. FtsZ is a tubulin ortholog that initiates cytokinesis by forming a ring structure at the cell centre. FtsZ recruits further proteins that eventually lead to the formation of a septum between the separated sister chromosomes [14,15]. In *E*. *coli*, proteins are assembled in a rather linear pathway [16], while in *B*. *subtilis*, a time delay exists between early recruited proteins (such as FtsA and ZapA) and late division proteins (such as FtsL and DivIb), indicating that proteins are recruited as complexes rather than singly [17]. Late division proteins include penicillin-binding proteins (Pbps) that synthesize the cell wall between the daughter cells. For growth as rods, actin-like MreB proteins are essential in many bacteria, interacting with Pbps and other membrane proteins involved in cell wall synthesis [18,19]. According to one theory, MreB forms filamentous structures underneath the cell membrane that direct the incorporation of new cell wall material via an interaction with the synthetic enzymes. The depletion of MreB leads to the generation of round cells that eventually lyse [20], showing that the protein plays an important function in cell shape maintenance.

Eukaryotic and prokaryotic membranes contain an asymmetric distribution of lipids. Especially cholesterol and sphingolipids in eukaroytes cluster into so called lipid rafts [21]. These dynamic microdomains also cluster proteins, many of which are involved in the transport of membrane components and in signal transduction. Flotillins are a class of membrane proteins that are associated with lipid rafts [22,23], but their detailed function is unclear. Flotillins are characterized by the SPFH domain of unknown function and extended heptad repeat regions. Recently, flotillin-like proteins FloT and YqfA have been implicated in the clustering of a signal transduction protein in the membrane of *B*. *subtilis* cells [24], revealing yet another striking parallel between pro – and eukaryotic cells.

In our work, we show that *B*. *subtilis* dynamin ortholog (termed DynA) plays a role in cell division. DynA and flotillin-like protein FloT synergistically affect cell division and cell morphology, suggesting that lipid raft formation and dynamin-driven membrane modification are important for cytokinesis and cell shape maintenance in bacteria.

## Results

### DynA plays a role in cell division

**Figure 1:**
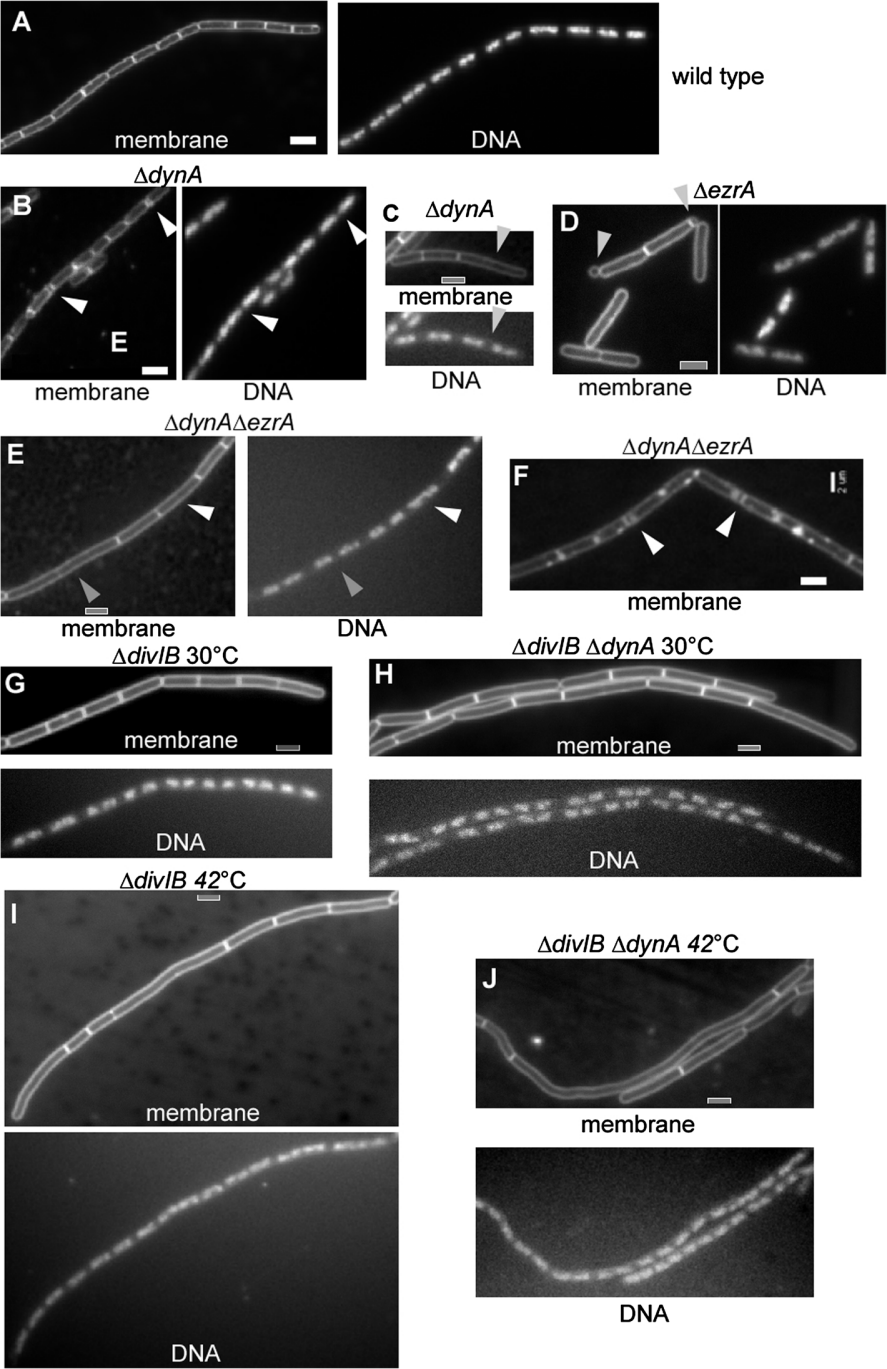
**Phenotypes of exponentially growing wild type (PY79) or mutant *Bacillus subtilis *cells. A**) Wild type cells, **B**) *dynA* (*ypbR*) mutant cells, white triangles indicate double septa, **C**) *dynA* (*ypbR*) mutant cells, grey triangle indicates highly elongated cell, **D**) *ezrA* mutant cells, **E**) *ezrA*/*dynA* double mutant cells, **F**) *ezrA*/*dynA* double mutant cells, white triangles indicate double septa, **G**) *divIB* mutant cells grown at 30°C, **H**) *divIB*/*dynA* double mutant cells grown at 30°C, **I**) *divIB* mutant cells grown at 42°C, **J**) *divIB*/*dynA* double mutant cells grown at 42°C. White or grey bars 2 μm.

We deleted the *dynA* (*ypbR*) gene by long flanking sequence homology PCR, such that only the first and last 100 bp of the gene remained within the chromosome, disrupted by a *tet* cassette. We also generated a truncated version of *dynA* through the insertion of a plasmid into the *dynA* gene, driving the downstream gene with a xylose-inducible promoter. Mutant cells grew with a doubling time indistinguishable from that of wild type cells, and contained nucleoids of normal appearance (Figure 1B). However, 5.5% of the cells showed double septa/mini cells (Figure 1B), which are never observed in wild type cells (Figure 1A). Additionally, 2.5% of mutant cells were larger than 5.5 μm (Figure 1C), while only 0.5% of wild type cells reach this size (250 cells measured for each strain). In contrast to e.g. a deletion of *sftA*, encoding for a DNA translocase that couples late states of chromosome segregation and cell division [25,26], DNA was never observed to be trapped in a closed division septum in *dynA* mutant cells. Therefore, chromosome segregation occurs normally in the mutant cells, but cell division is noticeably defective.

We wished to investigate the effect of a combination of the *dynA* deletion with that of a protein known to be important for an initial step in cell division. EzrA is a regulator of FtsZ, and therefore acts at a very early time point during cell division. The deletion of *ezrA* leads to the generation of elongated cells, to the formation of double septa and mini cells in rich medium [27]. In minimal medium used in this study, *ezrA* mutant cells were elongated, and formed mini cells (9%), but did not show any double septa (Figure 1D). Interestingly, *ezrA dynA* double mutant cells were more elongated than *ezrA* single mutant cells (Figure 1E), and contained more double septa than both single mutants (Figure 1F). Double mutant cells measured on average 5.16 ± 0.5 μm versus 4.07 ± 0.45 μm for *ezrA* mutant cells, and contained double septa in 15% of the cells versus 5% in *dynA* single mutant cells (with 200 cells measured for each strain from 2 independent experiments). Occasionally, long *ezrA dynA* double mutant cells showed a single condensed or decondensed nucleoid indicating a segregation defect, but this referred only to a subpupulation of long cells (Figure 1E, white triangle). Thus, the increase in cell length is largely due to an effect on cell division. These data suggest that EzrA and DynA affect two distinct steps early in cell division, each of which contributes to efficient cell division, because all phenotypes are exacerbated by the loss of both proteins.

We also tested if the *dynA* deletion is affected by the deletion of a gene involved in a later step of cell division. We used *divIB* mutant cells, which show a pronounced defect in cell division when they are shifted from 30 to 42°C. DivIB is a component of the DivIC/FtsL complex, which is recruited to the Z ring with a marked delay to the initial Z ring formation [17,28,29]. Indeed, when *divIB* mutant cells were shifted to the higher temperature, cells elongated markedly (compare Figure 1G and 1I), which was also true for *dynA divIB* double mutant cells, whose length could not easily be distinguished by eye from the *divIB* single mutant strain, neither at 30°C (Figure 1H) nor at 42°C (Figure 1J). We measured average cell length for 140 to 150 cells for each strain and for each growth temperature, from 3 independent experiments. The average cell length of *divIB* mutant cells was 4.03 μm (1.4 μm standard deviation, SD) at 30°C and 5.15 μm (4.9 μm SD) at 42°C, while that of *dynA divIB* mutant cells was 3.9 μm (1.2 μm SD) at 30°C and 6.18 μm (5.15 μm SD) at 42°C. Average cell length of *dynA* mutant cells at 42°C was 3.75 μm (1.1 μm SD). The high standard deviation at 42°C stems from the fact that a considerable number of cells were extremely long (up to 25 μm), while most cells had a size below 5 μm. To account for this, we grouped cells into three categories: cells below 5.5 μm, cells between 5.5 and 10 μm, and cells above 10 μm. For *divIB* single mutant cells, 6.3% of the cells were above 5.5 μm long, and 0.7% above 10 μm at 30°C, while at 42°C, 19% were above 5.5 μm and 8% above 10 μm. At 30°C, 8.5% of double mutant cells were above 5.5 μm and 1.5% above 10 μm, and at 42°C, 34% were above 5.5 μm and 12% above 10 μm (Table 1). Thus, the fraction of double mutant cells was higher in each of the “large cell” categories compared with the single *divIB* mutant cells. Single and double mutant cells contained normally segregated nucleoids (Figure 1G-J), showing that cell elongation is not an effect of delayed or blocked chromosome segregation. These data show that the deletion of a late cell division gene also exacerbates the *dynA* phenotype, showing that DynA does not only affect a step in cell division that is specific to the activity of EzrA.

**Table 1:** Distribution of cell length in single and double mutant cells

	<**5**.**5 μm**	>**5**.**5 μm** <**10 μm**	>**10 μm**
Δ*divIB* 30°C	93%	6.3%	0.7%
Δ*dynA* Δ*divIB* 30°C	90%	8.5%	1.5%
Δ*divIB* 42°C	73%	19%	8%
Δ*dynA* Δ*divIB* 42°C	64%	34%	12%

### DynA co-localizes with FtsZ and affects the formation of the Z ring

**Figure 2:**
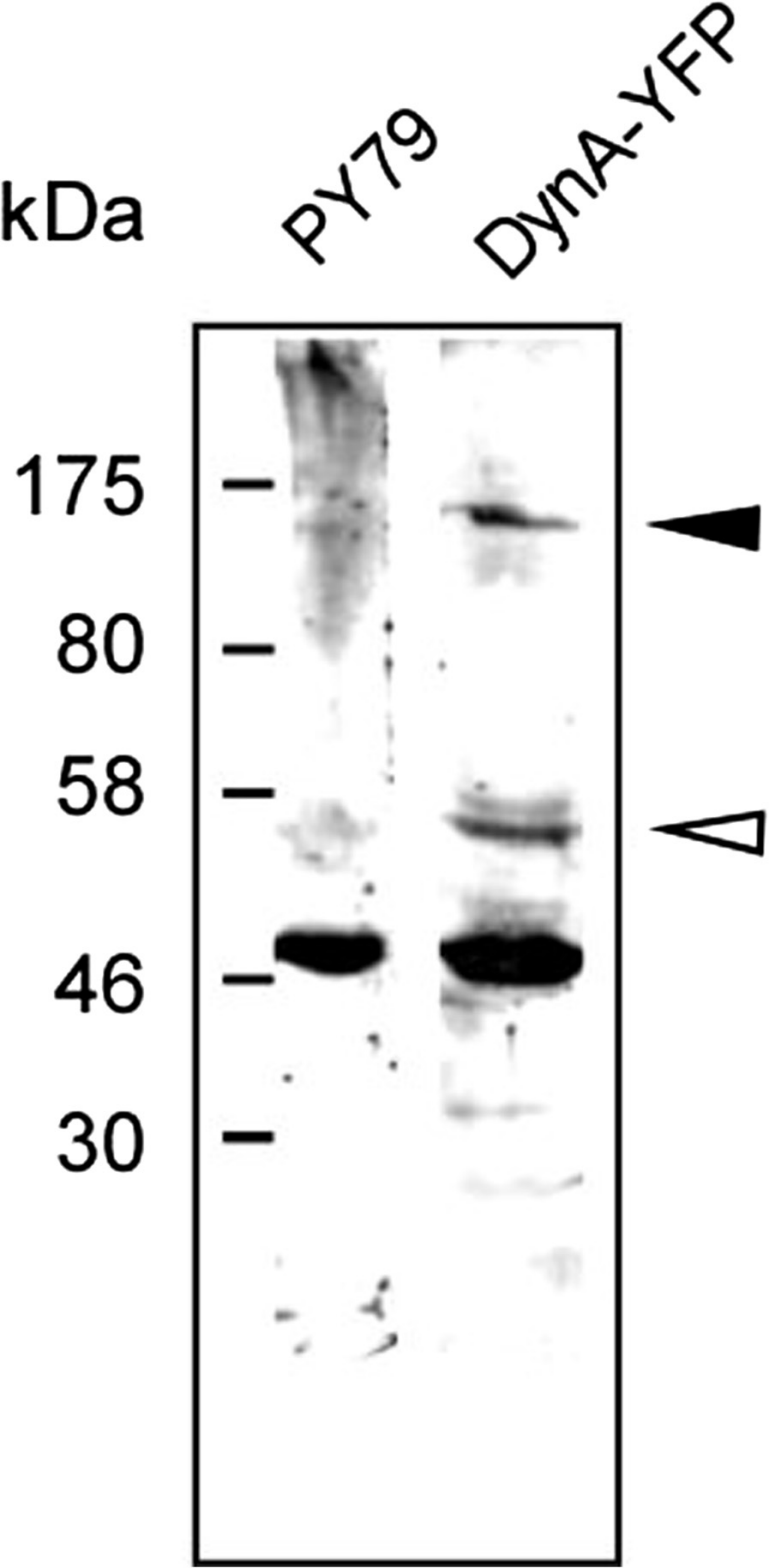
**Western blot of exponentially growing cells expressing DynA** (**PY79**) **or DynA**-**YFP as indicated above the lanes, using anti GFP antiserum.** Filled triangle corresponds to full length DynA-YFP, open triangle a C-terminal 27 kDa fragment of DynA plus YFP. Note that the band at 50 kDa is a crossreaction seen with the serum.

We generated a *dynA*(*ypbR*)-*yfp* fusion that was integrated into the original gene locus. Cells expressing DynA-YFP did not show any double septa, or highly elongated cells, indicating that the fusion can functionally replace the wild type protein and/or any of the possible post-translationally modified versions of DynA. Western blot analysis showed that full length DynA-YFP is expressed at extremely low levels, as well as a C-terminal fragment of 27 kDa and several smaller fragments (Figure 2, note that YFP is 28 kDa, giving rise to a band of 55 kDa). The 27 kDa band appeared at roughly the same level as full length protein. It is unclear if DynA is subject to rapid degradation through proteolysis or if the protein is proteolytically processed. Processing of DynA into two dynamin-like proteins (it consists of two fused dynamin modules) would give rise to 62 to 63 kDa sized proteins, which would be 90 kDa when fused to YFP. This is not the case according to the Western blot analysis. It is unclear if the truncation product is generated through the YFP fusion construct, or also occurs for wild type DynA. Therefore, localization studies must be viewed in light of the caveat that the truncation product may confer some level of DynA activity.

**Figure 3:**
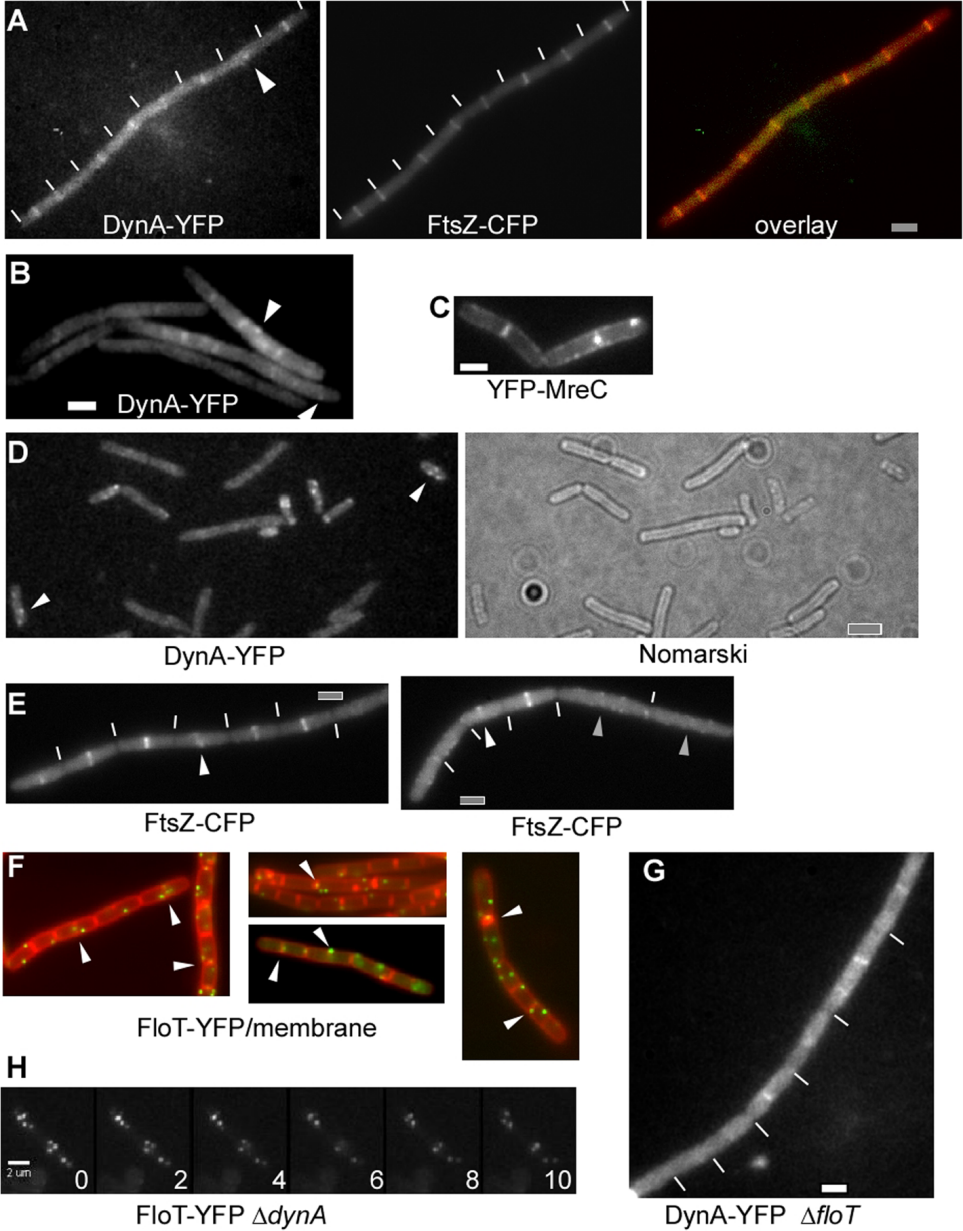
**Localization of DynA, FtsZ, FloT and MreB. A**-**B**) Growing wild type cells expressing DynA-YFP and FtsZ-CFP, white lines indicate septa between cells, overlay: FtsZ-CFP in red, DynA-YFP in green, **C**) cells expressing YFP-MreC, **D**) stationary phase cells expressing DynA-YFP, white triangles indicate membrane-proximal foci, **E**) *dynA* (*ypbR*) mutant cells expressing FtsZ-CFP, white triangles indicate asymmetric FtsZ rings, grey triangles large cells lacking FtsZ rings but instead containing membrane-proximal accumulations of FtsZ-CFP: white lines indicate septa between cells, **F**) wild type cells expressing FloT-YFP, overlay with membranes (red) and FloT-YFP (green), **G**) *floT* mutant cells expressing DynA-YFP. **H**) *dynA* mutant cells expressing FloT- YFP, time lapse with images taken every 2 s. White or grey bars 2 μm.

DynA-YFP localized to the cell center in exponentially growing cells (Figure 3A), and formed one or two foci at irregular places along the membrane in 15% of the cells (Figure 3B, 200 cells analyzed). Thus, in contrast to e.g. the membrane protein MreC, which localises as distinct foci throughout the membrane (Figure 3C, note that there are two adjacent membranes at the division septum), DynA is clearly highly enriched at the future division site. Indeed, DynA-YFP co-localized with FtsZ-CFP (Figure 3A); clear DynA-YFP fluorescence was seen at 85% of FtsZ-CFP rings, and 15% of Z rings were devoid of detectable DynA-YFP fluorescence (250 cells analysed), which, however, was extremely faint. Many cells contained DynA-YFP foci rather than ring-like structures (Figure 3A, indicated by white triangle). These data indicate that DynA is recruited to the Z ring, possibly at an early time point during cell division. 

During stationary phase, many cells showed multiple DynA-YFP foci, while most cells (60%) did not reveal any focus (Figure 3D). The observed foci were generally proximal to the cell membrane (Figure 3D), suggesting that DynA is largely a membrane-associated protein, although no obvious transmembrane segment is predicted by standard secondary structure prediction. Thus, DynA is associated with the cell division machinery in growing cells, in agreement with the observed phenotype of the *dynA* deletion, and remains membrane-associated in non-growing cells.

The apparent effect on cytokinesis prompted us to study the localization of FtsZ in *dynA* mutant cells. Although Z rings were normally positioned at mid cell in most *dynA* mutant cells, several abnormal morphologies of Z rings were observed: a) Z rings that appeared to be an open helix (Figure 3E, left panel), b) Z rings that were brighter on one side (Figure 3E, right panel), c) double septa (not shown) and d) missing rings in very large cells (> 4 μm, Figure 3E, right panel), which in wild type cells invariably contain Z rings. These aberrant structures were seen in about 15% of *dynA* mutant cells (180 cells analysed), indicating that DynA has an effect on the formation of a proper FtsZ ring, directly or indirectly, and that the defect in cell division arises largely through the loss of this function.

### A synthetic defect in cell division, cell shape maintenance and motility for dynamin and flotillin double mutant cells

**Figure 4:**
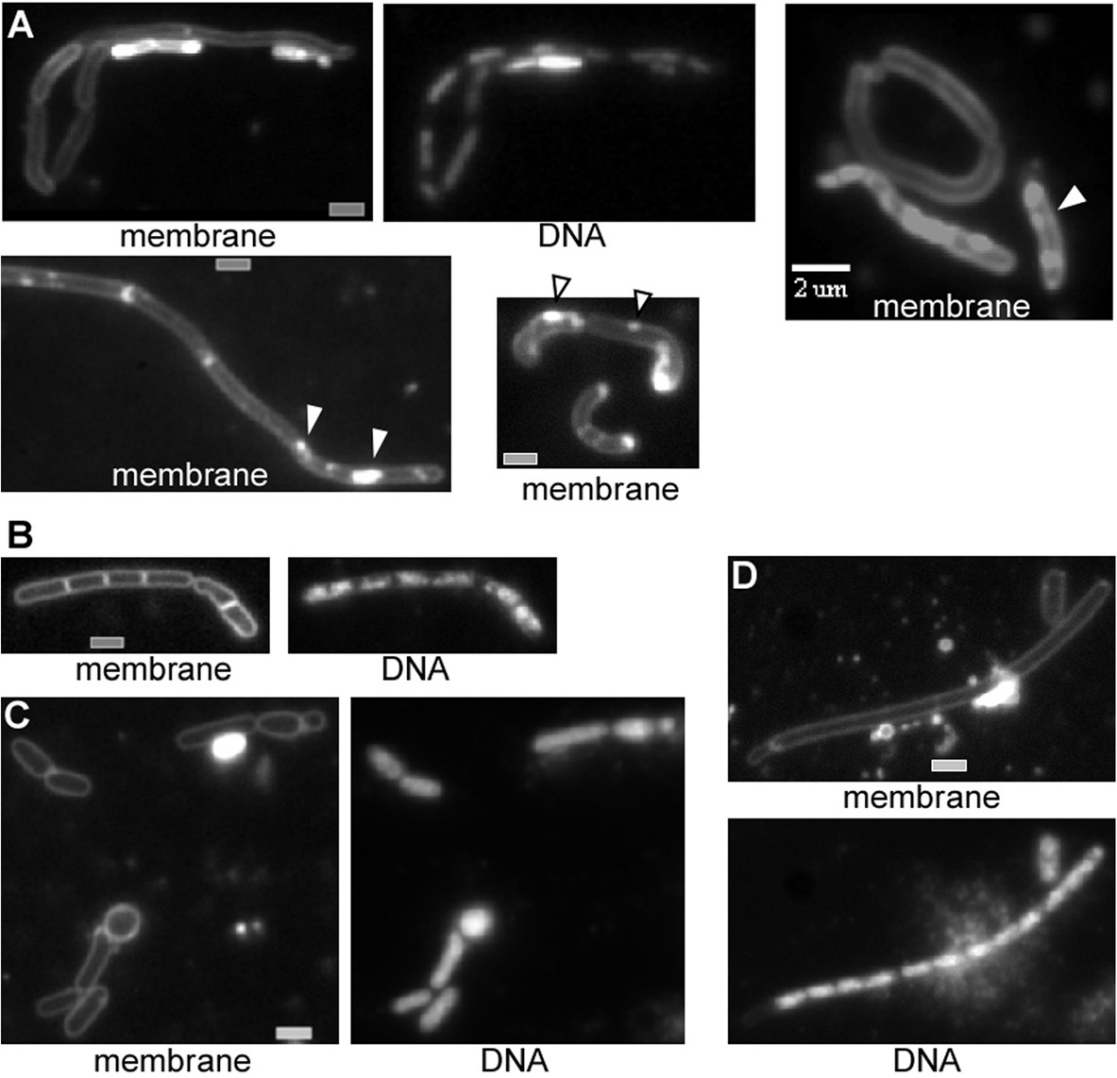
**Phenotypes of exponentially growing double mutant *B. subtilis *cells. A**) *dynA*/*floT* double mutant cells (note that membrane staining is highly heterogeneous between cells), white triangles indicate membrane abnormalities, **B**) *mreB* mutant cells grown in high magnesium medium, **C**-**D**) *dynA*/*mreB* double mutant cells growing in high magnesium medium. White or grey bars 2 μm.

**Figure 5:**
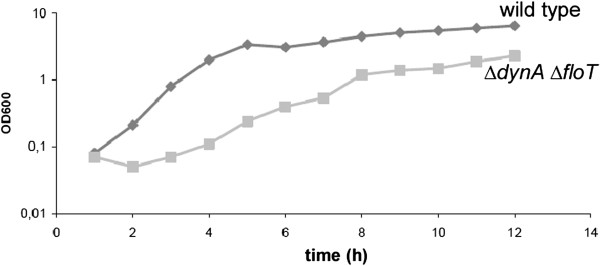
**Growth curve of wild type cells (diamonds) or of *dynA/floT *double mutant cells (squares) growing in S750 minimal glucose medium containing 0**.**1%casamino acids at 37°C.** Data are means from four independently growing cultures.

Eukaryotic membranes appear to have an asymmetric distribution of lipids, and specific proteins associated with the so-called lipid rafts. Flotillins are a divergent membrane protein family associated with lipid rafts, and are characterized by the SPFH domain of unknown function and extended heptad repeat regions [30]. *B*. *subtilis* flotillin-like proteins FloT and YqfA are involved in the clustering of a signal transduction protein in the membrane [24], and in the timing of initiation of sporulation [31]. Eukaryotic flotillin proteins are involved in clathrin-independent endocytosis, and in other processes, where membrane bending is of importance [32]. We reasoned that lipid rafts and bacterial dynamin may synergistically facilitate cell division, and therefore combined *floT* and *dynA* deletions. Strikingly, double mutant cells were highly elongated and showed a strong defect in cell shape maintenance (Figure 4A). Many cells were bent and had an irregular width, and a considerable fraction could reach a size of 12 μm. Frequently, cells showed aberrant membrane staining (Figure 4A), including large membrane perturbations. Although nucleoids were irregularly positioned, we did not observe any anucleate cells. In contrast to an *smc* mutant strain, in which chromosomes are highly decondensed and fill the entire cytoplasm (in which nucleoid occlusion blocks cell division [33]), *floT*/*yprB* double mutant cells contained many DNA-free sites in which nucleoid occlusion would not block division. However, cells were highly filamentous, suggesting that FloT and DynA synergistically affect cell division, in addition to an effect on rod-shape cell elongation. In agreement with the cytological data, the double mutant strain grew much slower than the wild type, and had a highly extended lag phase (Figure 5). Double mutant cells grew with a doubling time of 73 min versus 44 min at 37°C for wild type cells or for both single mutant strains, showing that growth was strongly compromised by the loss of both membrane-associated proteins. 

Based on its ability to tubulate membranes *in vitro*[11,13], DynA may facilitate membrane invagination through a mechanical bending of the membrane, while FloT may be important to generate a local environment favoring membrane curvature and/or recruitment of cell division proteins. In agreement with its function in lipid raft formation, a functional FloT-YFP fusion formed many discrete foci at the cell membrane [34] (Figure 3F). FloT-YFP was previously shown to move along random paths within or adjacent to the membrane [34]. These findings imply that due to the random movement, FloT would also be frequently present at mid cell, which indeed was shown to be the case by colocalization of FloT-YFP with membrane stain FM4-64 [34].To obtain a better idea about the extent of colocalization of FloT with the septal membrane, we quantified the number of FloT-YFP foci between cells. Indeed, 26% of FloT-YFP foci colocalized with the septal/polar membrane (184 foci analysed), or in other words, 22% of the cells had FloT-YFP fluorescence at the septum (Figure 3F, green FloT-YFP foci, red membrane) (148 cells analysed from 3 independent experiments), showing that FloT is present at sites of cell division in a large fraction of the cells; even more cells contained FloT-YFP foci close to the cell centre.

To investigate if one protein affects the localization of the other, we localized DynA-YFP in delta *floT* (*yuaG*) mutant cells. The localization pattern was indistinguishable from that of wild type cells (Figure 3G). Conversely, the absence of DynA did not visibly alter the localization pattern and dynamics of FloT-YFP (Figure 3H), showing that the proteins do not affect each other’s localization within the cell membrane and that they are not functionally linked.

### Synthetic phenotype of a *dynA mreB* double mutant strain

Because *floT dynA* double mutant cells had a highly disturbed cell shape, we investigated the effect of a *dynA* deletion in combination with an *mreB* deletion. MreB is essential for the maintenance of rod shape in many bacteria, and the depletion of MreB leads to the generation of round cells that eventually lyse [20,35]. However, *mreB* mutant cells can be grown in a medium containing a high concentration of magnesium [36], in which they show only a mild cell shape defect (Figure 4B). When grown under high magnesium conditions, a majority of *dynA mreB* double mutant cells showed a synthetic cell shape as well as division defect. A large fraction of cells was round or club-shaped, which was not observed for single mutant cells (Figure 4C). A second (smaller) fraction of cells was highly elongated (> 15 μm length), and many of these cells showed an irregular cell diameter along the length of the filaments (Figure 4D). In contrast to *dynA floT* double mutant cells, *dynA mreB* double mutants did not show membrane-abnormalities, indicating that these occur specifically due to the loss of dynamin and flotillin-like proteins, and not to a general alteration of cell morphology. Many *dynA mreB* double mutant cells contained decondensed chromosomes, but also contained segregated nucleoids, between which no septum was detectable, in spite of the excessive length of the cells (Figure 4D). In total, more than 90% of all double mutant cells showed a cell shape defect, while only 18% of the *mreB* single mutant cells showed a clear change in cell morphology (280 cells analysed). Therefore, DynA also plays a role in cell shape maintenance that is exacerbated by the loss of MreB.

To find out if DynA may have an effect in the formation of MreB filaments, as it has on the formation of the FtsZ ring, we visualized YFP-MreB in *dynA* mutant cells. Indistinguishably from wild type cells, YFP-MreB formed filamentous structures in mutant cells, which showed wild type-like remodeling (data not shown), showing that DynA itself does not directly affect the MreB cytoskeleton.

### Self assembly of DynA and of FloT at the membrane in a heterologous cell system

**Figure 6:**
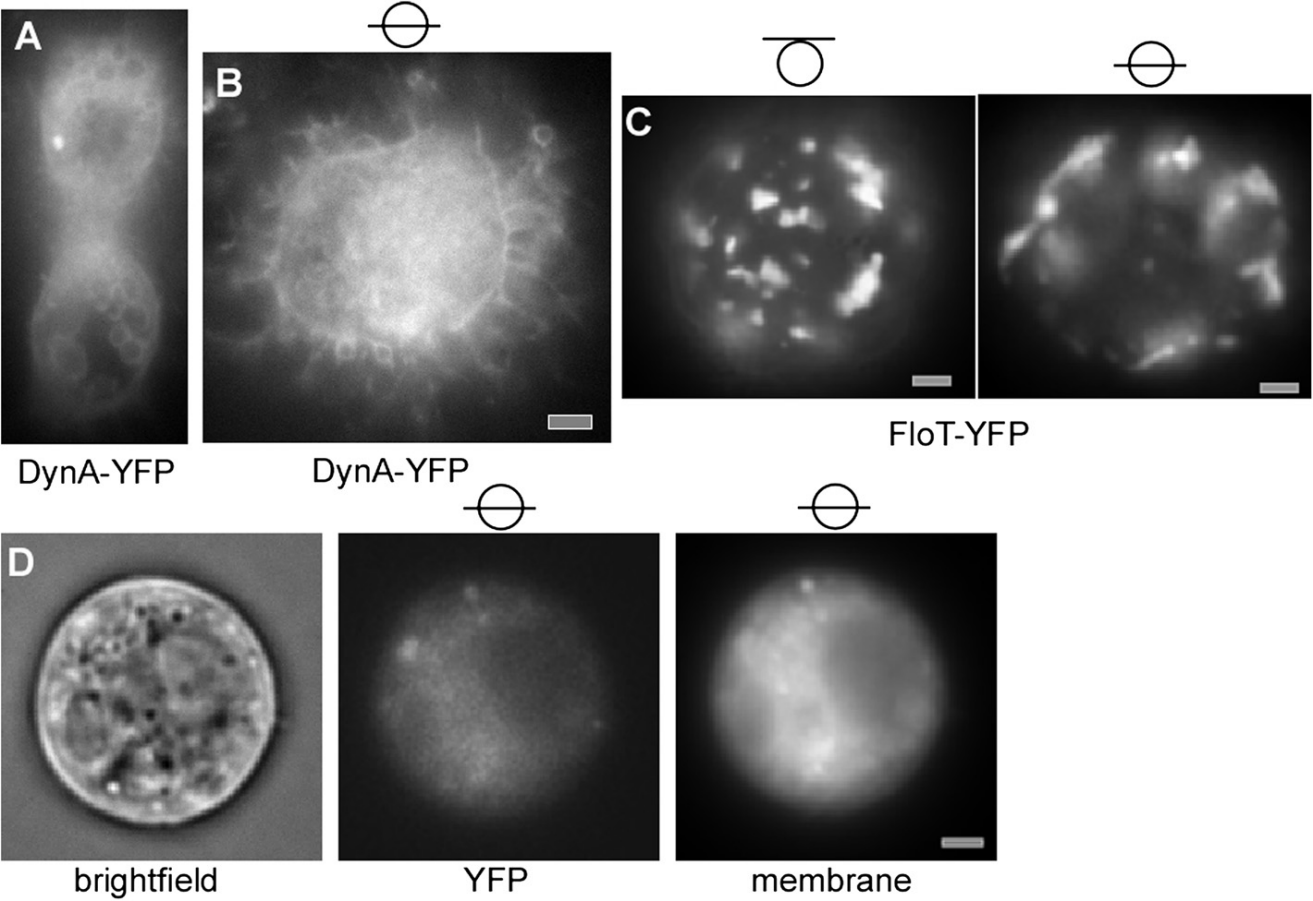
**YFP fluorescence of *Drosophila *S2 cells expressing fusion proteins. A**) cells expressing DynA-YFP early after induction, or **B**) 6 hours after induction. Shown are planes in the middle of cells, **C**) S2 cells expressing FloT-YFP, shown is the middle plane or the surface of the cells, as indicated by the lines within the circle. **D**) Non-transfected cells, the outline can be seen in the bright field channel; membrane stain also shows the outline of cells, but the membrane cannot be distinguished from the background of the cell; panel “YFP” shows background fluorescence in non-transfected cells in the YFP channel. White or grey bars 2 μm.

We wished to obtain information on the intrinsic properties of DynA, and therefore expressed the YFP fusion protein in Schneider S2 cells. These cells from *Drosophila* flies are highly diverged from the bacterial system, and because DynA displays less than 20% sequence identity with dynamin, it is highly unlikely that DynA has any specific interactors in S2 cells, or interacts with dynamin itself. Early after transfection, DynA-YFP assembled at internal membrane systems as well as underneath the cell membrane, suggesting that it has intrinsic membrane affinity (Figure 6A). After extended expression (6 hours and longer), DynA formed network-like structures at the cell membrane (note that membrane staining does not clearly show the outline of the membrane due to a high internal background, see Figure 6D). These structures resembled tubulated membrane structures, which extended away from the cells (Figure 6B). Non-transfected cells showed tubular membrane extensions at an about 100 fold lower level and only background fluorescence (Figure 6D), and started to form cell extensions 12 hours after deposition on glass slides (a time when they started to move), showing that DynA self assembles underneath the cell membrane and considerably distorts the membrane. In our model, we predict that dynamin distorts the cell membrane inwards during cell division, which is opposite from the orientation of the tubules observed in S2 cells. However, directionality of membrane distortion may be directed by other bacterial factors (e.g. by FtsZ), and tubules may also be caused by overproduction of DynA. In any event, our experiments show that DynA has the ability to induce considerable membrane distortion.

In contrast to DynA, FloT assembled only infrequently at internal membrane systems (occasionally, FloT-YFP was found around the nucleus) but predominantly at the cell membrane (Figure 6C), where it formed differently sized patch structures, as previously reported [34]. Given that FloT has extended coiled coil structures, we cannot exclude that the protein non-specifically interacts with other proteins within the membrane. However, usually, coiled coil interactions are rather specific, so our data indicate that FloT may self-assemble into raft-like structures in a heterologous system that lacks any other bacterial protein. FloT-YFP expressing cells showed very few tubulated membrane structures, verifying that DynA induces strong membrane deformation.

## Discussion

Bacterial dynamin-like proteins (BDLPs) have been characterized *in vitro*, and based on their ability to generate membrane tubulation and membrane fusion *in vitro*, a role in membrane dynamics [12], e.g. in late steps in cell division [13], has been proposed. However, it has been unclear if BDLPs confer any important role on the physiology of the cell. Through the combination of a *dynA* deletion with deletions in two genes involved in cell division, we show that indeed, DynA confers a function during cell division. A single *dynA* deletion leads to a very mild defect in Z ring formation, similar to, but less pronounced than, a deletion in *ezrA*. This is in agreement with our data showing that DynA colocalizes with FtsZ. 85% of the Z rings showed DynA-YFP signals (and because of the very weak fluorescence, the actual number could be higher). It has been shown that during spore germination, proteins such as EzrA and FtsA are recruited to the Z ring during the onset of division, while some proteins (such as DivIc and DivIb) are recruited with a 10 min time delay [17]. Assuming that this delay also applies to exponentially growing cells and not only to germinating cells, DynA might belong to the early cell division proteins, which must be verified by additional experiments. A *dynA ezrA* double deletion leads to a strongly exacerbated phenotype in cell division, suggesting that like EzrA, a regulator of FtsZ ring formation, *B*. *subtilis* dynamin affects an early stage in cell division. However, the combination of a *dynA* deletion with a *divIB* deletion also leads to a synthetic effect on cell division. DivIB affects a state in division clearly later than the formation of the Z ring, indicating that the function of DynA in division cannot be correlated with a defined stage in division. In any event, the accumulation of dynamin at the Z ring underlines the idea that dynamin confers a function during division.

Expression of DynA in a eukaryotic cell system showed that the protein has intrinsic affinity to the cell membrane and can assemble into tubulated structures. However, these pointed outwards of the cells, while the assumed function of dynamin in the bacterial cell would either be an inward bending of the membrane during cell division, or the fusion of membranes as the last step during division. It is likely that DynA needs cofactors for its appropriate function in the bacterium.

Interestingly, the combination of a *dynA* deletion with the deletion of a gene encoding for a flotillin-like protein, FloT, also leads to a synthetic defect in cell division. Flotillin proteins are implicated in lipid raft formation in eukaryotic and in prokaryotic cells. Although our experiments do not allow us to make any clear conclusion as to the detailed function of dynamin or flotillin, they show that bacterial dynamin and flotillin proteins play non-redundant functions in membrane dynamics. This is supported by our findings that each mutation does not affect the localization of the other protein. We suggest that dynamin is important for the generation of cell curvature, possibly via its putative mechanochemical activity, and likewise flotillin proteins, which may be important to recruit lipids that favour membrane bending. Indeed, there appears to be a link between flotillin in *B*. *subtilis* and membrane fluidity [37]. This idea is supported by our finding that DynA can distort the cell membrane in a heterologous cell system, suggesting that DynA may facilitate membrane invagination and/or couple Z-ring formation with membrane invagination. Alternatively, flotillin may be important to facilitate the recruitment of cell division proteins to the Z ring. In any event, the role of dynamin and flotillins in cell division is not redundant, because of the synthetic effect, and because of their different localization patterns.

The idea that dynamin and flotillin play general roles in membrane dynamics, rather than a role specific for cell division, is supported by three additional findings in our work: firstly, dynamin/flotillin double mutant cells grow much slower than wild type or single mutant cells, while cells depleted for FtsZ initially grow as fast as wild type cells. Thus, additional effects on cell growth are apparent in double mutant cells. Secondly, *dynA floT* double mutant cells also show a strong defect in cell morphology, and thirdly, the lack of the cytoskeletal element MreB in addition to the loss of dynamin function exacerbates the MreB cell shape phenotype. MreB can be deleted in the presence of high concentrations of magnesium (but not in normal medium), and the deletion of *dynA* under these conditions leads to a complete loss of rod cell morphology. Thus, dynamin function is also important in the context of maintenance of rod cell shape. DynA is not epistatic with MreB, showing that DynA does not act on cell morphology via the MreB cytoskeleton. Using fluorescence microscopy, we clearly identified DynA molecules along the lateral cell wall, away from the cell centre, which may be involved in functions affecting cell morphology.

## Conclusion

*In toto*, in our work, we uncover a role for DynA, the *Bacillus subtilis* ortholog of eukaryotic dynamin and of cyanobacterial BDLP, in cell division and in cell shape maintenance, and reveal a genetic link between bacterial dynamins and flotillins. We provide evidence that dynamin can self-assemble at the membrane and lead to membrane distortion in the absence of any bacterial cofactor. It is important to note that the lack of dynamin and of flotillin, or of dynamin and MreB, a gene involved in cell shape maintenance, results in various defects in the physiology of a bacterial cell, so the function of dynamin is not restricted to cell division. The data suggest that lipid rafts and dynamin-mediated membrane distortion play a synergistic role in a variety of membrane-associated assembly processes, the molecular nature of which needs to be further investigated.

## Methods

### Bacterial strains and media

*Bacillus* strains (Table 2) were grown in LB medium or, for microscopy, in S750 defined medium [38], which was complemented with 0.004% (w/v) casamino acids. Selection pressure with appropriate antibiotics was always kept when growing different strains. Cells were grown to exponential phase at 30°C. 

**Table 2:** Strains used in this study

**PY79**	**wt**	
HW2	*dynA*::*tet*	This study
HW3	*dynA*::*pMutin*	This study
FD249	*dynA*::*tet* Δ*floT*(in frame deletion)	This study
HW1	*dynA*-*yfp* (cm^R^)	This study
HW4	*dynA*-*yfp* (cm^R^) *ftsZ*-*cfp* (spec^R^)	This study
HW5	*dynA*::*tet ftsZ*-*cfp* (spec^R^)	This study
HW6	*dynA*::*tet yfp*-*mreB* (spec^R^)	This study
FD295	*floT*-*yfp* (cm^R^)	[34]
FD258	*dynA*::*tet floT*-*yfp* (cm^R^)	This study
3725	Δ*mreB* (in frame deletion)	[36]
HW7	*dynA*::*tet* Δ*mreB*	This study
HW8	*dynA*::*tet ezrA*::*spec*	This study
HIHO114	Δ*floT* in frame deletion	Gift from M. Hinderhofer
BS1059	P_xyl_*ftsZ*-*cfp* (spec^R^)	[39]
JS12	P_xyl_*yfp*-*mreB* (spec^R^)	[40]
FG375	*ezrA*::*spec*	[41]
Plasmids used for transfection		
pFD1	Expression vector for S2 cells	[42]
pFD239	*floT*-*yfp* in pFD1	[34]
pHW1	*dynA*-*yfp* in pFD1	This study

For the deletion of *dynA* (*ypbR*), the first 100 bp of *dynA* plus 900 bp upstream were amplified via PCR, and likewise the last 100 bp plus the following 900 bp. Inner primers contained 18 bp of homology to the tet cassette, which was amplified together with flanking sequences in a second PCR reaction. The resulting 4000 bp fragment was used for transformation of PY79 wild type cells, selecting for tetracycline (tet) resistance, giving rise to HW2 (*dynA*::*tet*). As an alternative strategy, 500 bp internal of *dynA* (starting at bp 1480) were amplified and cloned into pMutin, using *Hin*dIII and *Eco*RI restriction sites. PY79 cells were transformed with plasmid DNA, selecting for Mls resistance, giving rise to a DynA truncation missing the last 500 amino acids.

For the generation of a *dynA floT* double mutant strain, strain DML1541 Δ*floT* (*yuaG*) (in frame deletion of *yuaG*, kind gift from M. Hinderhofer, University of Konstanz) was transformed with chromosomal DNA from strain HW2, selecting for tet resistance. For the generation of a C-terminal YFP fusion to DynA, the last 500 bp of *dynA* were amplified by PCR and were closed into pSG1164YFP [43] using *Apa*I and *Eco*RI restriction sites. PY79 cells were transformed with the resulting plasmid, which integrated at the *dynA* locus via single crossover integration (this was verified by PCR using a pair of primers that binds within the *yfp* gene and upstream of the 500 bp used for integration). Expression of full length DynA-YFP was verified by Western blotting. For simultaneous visualization of DynA and of FtsZ, strain HW1 (DynA-YFP) was transformed with chromosomal DNA from strain BS1059 [39], in which FtsZ-CFP is expressed from a xylose inducible fusion at the amylase locus. The resulting colonies were obtained through selection on spectinomycin containing plates. For the localization of FtsZ-CFP or of YFP-MreB in *dynA* mutant cells, strain BS1059 or JS12 was transformed with chromosomal DNA from strain HW2, respectively.

To visualize FloT-YFP in the absence of DynA, strain HW2 (Δ *dynA*) was transformed with chromosomal DNA of strain FD295 (*floTyfp*). To create a *dynA mreB* double deletion, strain 3725 [36] (Δ*mreB*) was transformed with chromosomal DNA of strain HW2 (Δ *dynA*) and incubated at 25°C using PAB/SMM agar [44]. The *ezrA dynA* double deletion was created by transformation of strain HW2 (Δ*dynA*) with chromosomal DNA of strain FG375 (kind gift from F. Gueiros-Filho, University of São Paulo, Brasil).

The plasmids used for S2 cell transfection were created by cloning the complete coding sequence of DynA or of FloT into the vector pFD1 [45], using *Kpn*I and *Xho*I or *Apa*I and *Cla*I, respectively.

### Schneider cell culture and transient transfection

*D*. *melanogaster* S2 Schneider cells were grown in Schneider’s Drosophila medium (Lonza Group Ltd.) supplemented with 5-10% (v/v) fetal calf serum (FCS) at 25°C without addition of CO_2_. Cells were passaged every 2 to 3 days to maintain optimal growth. For transfection, S2 cells were spread in a 6-well plate at 1 × 10^6^ per well in 3 ml medium containing 5% FCS. Supercoiled plasmids (0.3 μg of each plasmid) were complexed with lipid (10 μl FuGENE HD reagent, Roche) in 200 μl serum-free medium. The complex was incubated at room temperature for 15 min, filled up with serum-free medium to 1 ml and then added to cells from which the growth medium was removed (cells were washed 1 × with serum-free medium). After 18 hrs, the complex suspension was removed and replaced by 3 ml of medium containing 10% (v/v) FCS. After further incubation for 24 h, the production of the proteins was induced by adding CuSO_4_ to a final concentration of 1 mM.

### Image acquisition

Fluorescence microscopy was performed on an Olympus AX70 microscope with a Cool Snap ES2 camera (Photometrics), TIRF microscopy was performed on an inverted Zeiss Axioobserver microscope with a TIRF incorporation from Visitron (Munich), and an Evolve EMCCD camera (Photometrics). Cells were mounted on thin agarose pads (1% w/v prepared in S7_50_ minimal medium) on an object slide. DNA was stained with 4^′^, 6-diamidino-2-phenylindole (DAPI; final concentration 0.2 ng/ml), membranes with FM4-64 (Molecular Probes). Images were processed with Metamorph software.

## Competing interests

There are no financial or non-financial competing interests concerning this publication. The article processing charge was funded by the German Research Foundation (DFG) and the Albert Ludwigs University Freiburg in the funding programme Open Access Publishing. The University does not gain any financially from this publication.

## Authors' contributions

FD generated genetic constructs and strains, performed most image acquisitions, evaluated data and helped writing the manuscript. HW generated genetic constructs and strains, and performed several microscopy experiments. FD and HW performed growth experiments. MS constructed strains concerning the *divIb* mutation and performed the related experiments. PLG conceived of the study and wrote the manuscript. PLG, FD, HW and MS evaluated data. All authors read and approved the final manuscript.

## References

[B1] Hinshaw JE Dynamin and its role in membrane fission Annu Rev Cell Dev Biol 2000 16 483 519 10.1146/annurev.cellbio.16.1.483 11031245 PMC4781412

[B2] Osteryoung KW Nunnari J The division of endosymbiotic organelles Science 2003 302 5651 1698 1704 10.1126/science.1082192 14657485

[B3] Low HH Lowe J Dynamin architecture-from monomer to polymer Curr Opin Struct Biol 2010 20 6 791 798 10.1016/j.sbi.2010.09.011 20970992

[B4] Praefcke GJ McMahon HT The dynamin superfamily: universal membrane tubulation and fission molecules? Nat Rev Mol Cell Biol 2004 5 2 133 147 10.1038/nrm1313 15040446

[B5] Song BD Schmid SL A molecular motor or a regulator? Dynamin's in a class of its own Biochemistry 2003 42 6 1369 1376 10.1021/bi027062h 12578348

[B6] Danino D Hinshaw JE Dynamin family of mechanoenzymes Curr Opin Cell Biol 2001 13 4 454 460 10.1016/S0955-0674(00)00236-2 11454452

[B7] Niemann HH Knetsch ML Scherer A Manstein DJ Kull FJ Crystal structure of a dynamin GTPase domain in both nucleotide-free and GDP-bound forms EMBO J 2001 20 21 5813 5821 10.1093/emboj/20.21.5813 11689422 PMC125706

[B8] Baba T Damke H Hinshaw JE Ikeda K Schmid SL Warnock DE Role of dynamin in clathrin-coated vesicle formation Cold Spring Harb Symp Quant Biol 1995 60 235 242 10.1101/SQB.1995.060.01.027 8824396

[B9] Pucadyil TJ Schmid SL Conserved functions of membrane active GTPases in coated vesicle formation Science 2009 325 5945 1217 1220 10.1126/science.1171004 19729648 PMC2864031

[B10] Sever S Damke H Schmid SL Dynamin: GTP controls the formation of constricted coated pits, the rate limiting step in clathrin-mediated endocytosis J Cell Biol 2000 150 5 1137 1148 10.1083/jcb.150.5.1137 10974001 PMC2175254

[B11] Low HH Lowe J A bacterial dynamin-like protein Nature 2006 444 7120 766 769 10.1038/nature05312 17122778

[B12] Low HH Sachse C Amos LA Lowe J Structure of a bacterial dynamin-like protein lipid tube provides a mechanism for assembly and membrane curving Cell 2009 139 7 1342 1352 10.1016/j.cell.2009.11.003 20064379 PMC2862293

[B13] Burmann F Ebert N van Baarle S Bramkamp M A bacterial dynamin-like protein mediating nucleotide-independent membrane fusion Mol Microbiol 2011 79 5 1294 1304 10.1111/j.1365-2958.2011.07523.x 21205012

[B14] Adams DW Errington J Bacterial cell division: assembly, maintenance and disassembly of the Z ring Nat Rev Microbiol 2009 7 9 642 653 10.1038/nrmicro2198 19680248

[B15] Rothfield L Taghbalout A Shih YL Spatial control of bacterial division-site placement Nat Rev Microbiol 2005 3 12 959 968 10.1038/nrmicro1290 16322744

[B16] Margolin W FtsZ and the division of prokaryotic cells and organelles Nat Rev Mol Cell Biol 2005 6 11 862 871 10.1038/nrm1745 16227976 PMC4757588

[B17] Gamba P Veening JW Saunders NJ Hamoen LW Daniel RA Two-step assembly dynamics of the *bacillus subtilis* divisome J Bacteriol 2009 191 13 4186 4194 10.1128/JB.01758-08 19429628 PMC2698510

[B18] Pichoff S Lutkenhaus J Overview of cell shape: cytoskeletons shape bacterial cells Curr Opin Microbiol 2007 10 6 601 605 10.1016/j.mib.2007.09.005 17980647 PMC2703429

[B19] Graumann PL Cytoskeletal elements in bacteria Annu Rev Microbiol 2007 61 589 618 10.1146/annurev.micro.61.080706.093236 17506674

[B20] Jones LJ Carballido-Lopez R Errington J Control of cell shape in bacteria: helical, actin-like filaments in bacillus subtilis Cell 2001 104 6 913 922 10.1016/S0092-8674(01)00287-2 11290328

[B21] Lingwood D Simons K Lipid rafts as a membrane-organizing principle Science 2010 327 5961 46 50 10.1126/science.1174621 20044567

[B22] Browman DT Hoegg MB Robbins SM The SPFH domain-containing proteins: more than lipid raft markers Trends Cell Biol 2007 17 8 394 402 10.1016/j.tcb.2007.06.005 17766116

[B23] Langhorst MF Reuter A Stuermer CA Scaffolding microdomains and beyond: the function of reggie/flotillin proteins Cell Mol Life Sci 2005 62 19–20 2228 2240 16091845 10.1007/s00018-005-5166-4 PMC11139094

[B24] Lopez D Kolter R Functional microdomains in bacterial membranes Genes Dev 2010 24 17 1893 1902 10.1101/gad.1945010 20713508 PMC2932971

[B25] Kaimer C Gonzalez-Pastor JE Graumann PL SpoIIIE and a novel type of DNA translocase, SftA, couple chromosome segregation with cell division in *bacillus subtilis* Mol Microbiol 2009 74 4 810 825 10.1111/j.1365-2958.2009.06894.x 19818024

[B26] Biller SJ Burkholder WF The *bacillus subtilis* SftA (YtpS) and SpoIIIE DNA translocases play distinct roles in growing cells to ensure faithful chromosome partitioning Mol Microbiol 2009 74 4 790 809 10.1111/j.1365-2958.2009.06893.x 19788545

[B27] Levin PA Kurtser IG Grossman AD Identification and characterization of a negative regulator of FtsZ ring formation in *bacillus subtilis* Proc Natl Acad Sci USA 1999 96 17 9642 9647 10.1073/pnas.96.17.9642 10449747 PMC22263

[B28] Harry EJ Wake RG The membrane-bound cell division protein DivIB is localized to the division site in *bacillus subtilis* Mol Microbiol 1997 25 2 275 283 10.1046/j.1365-2958.1997.4581822.x 9282739

[B29] Daniel RA Noirot-Gros MF Noirot P Errington J Multiple interactions between the transmembrane division proteins of *bacillus subtilis* and the role of FtsL instability in divisome assembly J Bacteriol 2006 188 21 7396 7404 10.1128/JB.01031-06 16936019 PMC1636283

[B30] Hinderhofer M Walker CA Friemel A Stuermer CA Moller HM Reuter A Evolution of prokaryotic SPFH proteins BMC Evol Biol 2009 9 10 10.1186/1471-2148-9-10 19138386 PMC2636767

[B31] Donovan C Bramkamp M Characterization and subcellular localization of a bacterial flotillin homologue Microbiology 2009 155 Pt 6 1786 1799 19383680 10.1099/mic.0.025312-0

[B32] Glebov OO Bright NA Nichols BJ Flotillin-1 defines a clathrin-independent endocytic pathway in mammalian cells Nat Cell Biol 2006 8 1 46 54 10.1038/ncb1342 16341206

[B33] Wu LJ Errington J Coordination of cell division and chromosome segregation by a nucleoid occlusion protein in *bacillus subtilis* Cell 2004 117 7 915 925 10.1016/j.cell.2004.06.002 15210112

[B34] Dempwolff F Moller HM Graumann PL Synthetic motility and cell shape defects associated with deletions of flotillin/reggie paralogs in *bacillus subtilis* and interplay of these proteins with NfeD proteins J Bacteriol 2012 194 17 4652 4661 10.1128/JB.00910-12 22753055 PMC3415494

[B35] Defeu Soufo HJ Graumann PL Actin-like proteins MreB and Mbl from *bacillus subtilis* are required for bipolar positioning of replication origins Curr Biol 2003 13 21 1916 1920 10.1016/j.cub.2003.10.024 14588250

[B36] Formstone A Errington J A magnesium-dependent *mreB* null mutant: implications for the role of mreB in *bacillus subtilis* Mol Microbiol 2005 55 6 1646 1657 10.1111/j.1365-2958.2005.04506.x 15752190

[B37] Lee YH Kingston AW Helmann JD Glutamate dehydrogenase affects resistance to cell wall antibiotics in *bacillus subtilis* J Bacteriol 2011 194 5 993 1001 22178969 10.1128/JB.06547-11 PMC3294759

[B38] Jaacks KJ Healy J Losick R Grossman AD Identification and characterization of genes controlled by the sporulation regulatory gene *spo0H* in *bacillus subtilis* J Bacteriol 1989 171 4121 4129 2502532 10.1128/jb.171.8.4121-4129.1989 PMC210181

[B39] Feucht A Lewis PJ Improved plasmid vectors for the production of multiple fluorescent protein fusions in *Bacillus subtilis* Gene 2001 264 2 289 297 10.1016/S0378-1119(01)00338-9 11250085

[B40] Defeu Soufo HJ Graumann PL Dynamic localization and interaction with other *bacillus subtilis* actin-like proteins are important for the function of MreB Mol Microbiol 2006 62 1340 1356 10.1111/j.1365-2958.2006.05457.x 17064365

[B41] Gueiros-Filho FJ Losick R A widely conserved bacterial cell division protein that promotes assembly of the tubulin-like protein FtsZ Genes Dev 2002 16 19 2544 2556 10.1101/gad.1014102 12368265 PMC187447

[B42] Dempwolff F Reimold C Reth M Graumann PL *Bacillus subtilis* MreB orthologs self-organize into filamentous structures underneath the cell membrane in a heterologous cell system PLoS One 2011 6 11 e27035 10.1371/journal.pone.0027035 22069484 PMC3206058

[B43] Kidane D Sanchez H Alonso JC Graumann PL Visualization of DNA double-strand break repair in live bacteria reveals dynamic recruitment of *bacillus subtilis* RecF, RecO and RecN proteins to distinct sites on the nucleoids Mol Microbiol 2004 52 6 1627 1639 10.1111/j.1365-2958.2004.04102.x 15186413

[B44] Defeu Soufo HJ Reimold C Linne U Knust T Gescher J Graumann PL Bacterial translation elongation factor EF-Tu interacts and colocalizes with actin-like MreB protein Proc Natl Acad Sci USA 2010 107 7 3163 3168 10.1073/pnas.0911979107 20133608 PMC2840354

[B45] Waidner B Specht M Dempwolff F Haeberer K Schaetzle S Speth V Kist M Graumann PL A novel system of cytoskeletal elements in the human pathogen *helicobacter pylori* PLoS Pathog 2009 5 11 e1000669 10.1371/journal.ppat.1000669 19936218 PMC2776988

